# Renaming Palliative Cancer Therapies: Call It What It Is

**DOI:** 10.1093/oncolo/oyae039

**Published:** 2024-03-18

**Authors:** Kimberly E Kopecky, Toby C Campbell

**Affiliations:** Department of Surgery, Division of Surgical Oncology, University of Alabama at Birmingham, Birmingham, AL, USA; Division of Hematology/Oncology, Department of Medicine, University of Wisconsin-Madison, Madison, WI, USA

## Abstract

This commentary explores the language choices that oncology providers make when discussing cancer therapy goals.

A staggering 69% of patients receiving non-curative chemotherapy for metastatic lung cancer and 50% of patients receiving chemotherapy for advanced gastroesophageal, hepatobiliary, or pancreatic cancer reported that the goal of therapy was to cure their cancer.^1^ Similarly, 81% of patients with metastatic colorectal cancer believe that the chemotherapy they are receiving has curative potential.^2^ Why does this disconnect exist and what might we do about it?

While there are certainly a myriad of reasons for this misunderstanding, the language choices that oncology providers make when discussing cancer therapy goals are a fundamental domain to explore. To start, the word palliative is routinely used in discussions with patients and their families despite the fact that a majority of patients are unfamiliar with what the word palliative means.^3,4^ When ambiguous terminology is used as medical jargon (perhaps unintentionally), the noncurative intent of therapy is obscured and the need to communicate a bad prognosis can be avoided. For patients, the use of unclear language can propagate uncertainty regarding the indications for treatment and allow them to imagine that what they desperately hope for—cure—is actually a shared goal. We recommend a single-word substitution to call it what it is by using the word non-curative [therapy] in place of palliative [therapy] with patients and their families. The goal of this semantic change is to convey a clear and consistent message—this therapy is being given without the intent to cure.

Why does this matter? Before a patient can make decisions, set goals, or cope with the reality of a new cancer diagnosis, they need to first understand the recommended treatment plan and be able to recognize how it is and is not expected to alter the course of their disease. This information is particularly essential given that cancer treatment options are laden with side effects such as fatigue, nausea, neutropenia, neuropathy, anorexia, infection, and complications from surgery or other procedural interventions. When clinicians use clear language with patients and their families by using the words non-curative [therapy] in place of palliative [therapy], discussion is naturally facilitated about treatment expectations and goals. When the goals of a particular therapy are communicated clearly—be it to live longer, feel better, or prevent disability^5^—patients are empowered to consider the balance between anticipated side effects and the therapeutic benefit of alleged palliative interventions.^6^

Is there enough data to support the notion that non-curative cancer treatments universally alleviate symptoms caused by cancer? Unfortunately, no. The evidence regarding the therapeutic benefit of palliative-intent therapies in terms of quality of life and symptom management in the setting of advanced cancer is limited. Our best data suggest that non-curative systemic therapies most commonly delay the progression of symptoms and there is a lack of evidence that palliative procedural interventions universally alleviate pain and the burden of cancer-associated symptoms. Additionally, there is a gap in our understanding of which patients do and do not clinically benefit from these treatment options. In light of such limited data and the potential for treatment-related morbidity and mortality, it is misleading to assert that the majority of palliative-intent interventions reliably offer symptomatic benefit. Given that accurate expectations of treatment are required for patients to make choices that are in line with their goals, misperception of treatment efficacy and/or objective compromises the ability of patients to make informed decisions.

Fundamentally, a patient’s perception of their overall health, cancer-related disease factors, and prognostic understanding is multifaceted, and remodeling a physician’s communication practice is difficult. There are certainly additional moments where treatment intention might be discussed or to say, “this cancer is incurable.” We believe, however, that routine use of the word non-curative rather than palliative is accurate and clear while appropriately respecting patient autonomy regarding the disclosure of specific prognostic information.

It has been suggested that “structured communication can overcome the inherent ambiguity of the term ‘palliative chemotherapy’.”^7^ We advocate for a simpler approach: call it what it is (Fig. 1).

**Figure 1.**
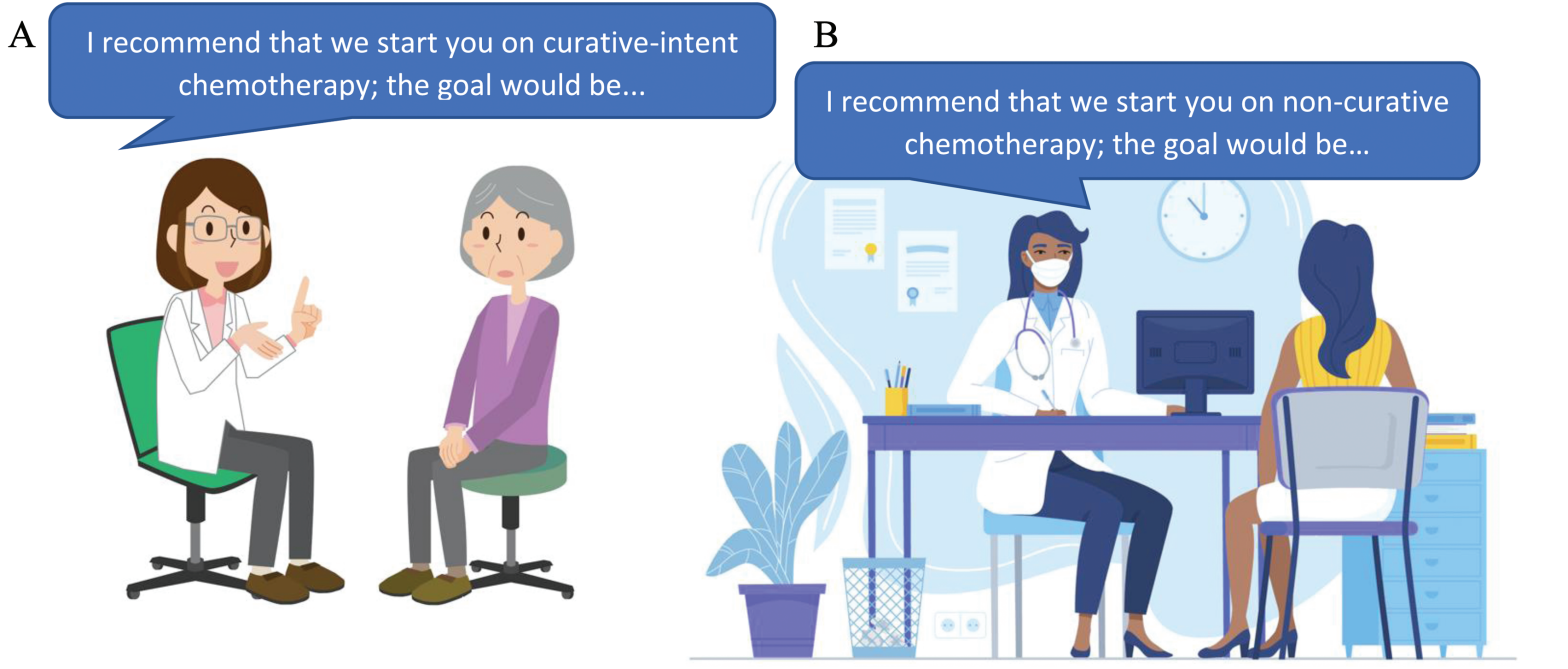
Clinicians speak with patients using the language outlined in the manuscript. In panel (**A**), the medical provider tells the patient “I recommend that we start you on curative-intent chemotherapy.” In panel (**B**), the medical provider tells the patient “I recommend that we start you on non-curative chemotherapy.” Figure license purchased from © Eezy Inc.

## References

[1] El-Jawahri A , Traeger L, Park ER, et al. Associations among prognostic understanding, quality of life, and mood in patients with advanced cancer: prognosis, QoL, and mood in advanced CA. Cancer. 2014;120(2):278-285. 10.1002/cncr.28369 24122784

[2] Weeks JC , Catalano PJ, Cronin A, et al. Patients’ expectations about effects of chemotherapy for advanced cancer. N Engl J Med. 2012;367(17):1616-1625. 10.1056/NEJMoa1204410 23094723 PMC3613151

[3] Bazargan M , Cobb S, Assari S, Kibe LW. Awareness of palliative care, hospice care, and advance directives in a racially and ethnically diverse sample of California adults. Am J Hosp Palliat Care. 2021;38(6):601-609. 10.1177/1049909121991522 33535787

[4] Pentz RD , Lohani M, Hayban M, et al. Videos improve patient understanding of misunderstood chemotherapy terminology. Cancer. 2019;125(22):4011-4018. 10.1002/cncr.32421 31418849 PMC6986301

[5] Schwarze ML , Kruser JM, Clapp JT. Innovations in surgical communication 2—focus on the goals of surgery. JAMA Surg. 2023;158(10):994-996. 10.1001/jamasurg.2023.3340 37556129 PMC11072112

[6] Doverspike L , Kurtz S, Selvaggi K. Palliative chemotherapy: does it only provide false hope? The role of palliative care in a young patient with newly diagnosed metastatic adenocarcinoma. J Adv Pract Oncol. 2017;8(4):382-386. 30018843 PMC6040871

[7] Wulff-Burchfield E , Spoozak L, Finlay E. Palliative chemotherapy and the surgical oncologist. Surg Oncol Clin N Am. 2021;30(3):545-561. 10.1016/j.soc.2021.02.008 34053668

